# Impact of Chronic Kidney Insufficiency on Cardiovascular Outcomes in Patients that Undergo Coronary Revascularization: A Historical Review

**DOI:** 10.7603/s40602-016-0008-1

**Published:** 2016-11-16

**Authors:** Koh Choong Hou, Kenny Sin Yoong Kong, Terence Kee Yi Shern, Jack Tan Wei Chieh

**Affiliations:** 1 Department of Cardiovascular Medicine, National Heart Centre Singapore, 5 Hospital Drive, Singapore, Singapore 169609; 2 Department of Cardiothoracic Surgery, National Heart Centre Singapore, 5 Hospital Drive, Singapore, Singapore 169609; 3 Department of Renal Medicine, Singapore General Hospital, Outram Road, Singapore, Singapore 169608

## Abstract

Chronic kidney disease (CKD) is associated with poorer short and long-term cardiovascular morbidity and mortality. Even after the commencement of haemodialysis in end stage renal failure patients, mortality exceeds 20% in the first year^1^. More than 50% of these deaths are contributed by cardiovascular diseases (CVD), of which 20% are caused by acute myocardial infarction^2^. Consequent to these findings, the degree and impact of coronary revascularization on CKD patients represents a clinical challenge, especially in the setting of advanced stages of CKD.

## Introduction

Chronic kidney disease (CKD) is associated with poorer short and long-term cardiovascular morbidity and mortality. Even after the commencement of haemodialysis in end stage renal failure patients, mortality exceeds 20% in the first year^1^. More than 50% of these deaths are contributed by cardiovascular diseases (CVD), of which 20% are caused by acute myocardial infarction^2^. Consequent to these findings, the degree and impact of coronary revascularization on CKD patients represents a clinical challenge, especially in the setting of advanced stages of CKD.

This paper attempts to review the recent literature and data on CKD and its corollary cardiovascular effects, and the implications of different revascularization strategies on both short and long-term clinical outcomes. While percutaneous coronary intervention (PCI) techniques, instruments and pharmacotherapeutics had progressively advanced in a leap frogging fashion over conventional surgical coronary bypass, it has yet to demonstrate superior outcomes in the CKD population. This review aims to look at the latest evidence in the evolution of PCI, and whether this has led to improved CVD outcomes in CKD patients.

## Methods

MEDLINE, EMBASE and clinicaltrials.gov databases were searched to identify relevant studies. Studies that reviewed or compared clinical outcomes of coronary revascularization were included. Key words included: chronic renal insufficiency, dialysis, coronary revascularization, coronary artery bypass surgery, percutaneous coronary intervention and/or angioplasty, drug eluting stent, bare metal stent.

Both randomized and non-randomized studies were included. In total, 56 citations were reviewed from the online databases, and 4 citations reviewed through the reference lists. Twentythree citations were excluded based on title and abstract review using the inclusion key words, with 37 references included in the final review.

## Background

In the setting of CKD, the development of CVD is accelerated, as are the adverse outcomes of atherosclerotic CVD (ASCVD). It had already been previously noted that CKD had the following relationships and effects on the cardiovascular system:

1. The severity and incidence of CAD worsens as renal function declines^3^,^4^, with the prevalence of triple vessel disease or left main CAD being significantly higher in the CKD population.
2. CKD patients tend to develop multivessel CAD with significant coronary calcifications, this in turn is strongly associated with exercise induced ischemia, as observed by stress echocardiographic studies^5^.
3. Among patients with CAD, the presence of CKD portends a more adverse cardiovascular prognosis^6^. One small study^7^ observed that significant epicardial coronary stenoses of >50% luminal diameter were already present prior to renal replacement therapy (RRT) initiation, and that such stenoses were often missed by stress perfusion imaging modalities.
4. Standard cardiovascular risk factors (CVRF) are common in both CKD and CAD. subgroups^8^. Dialysis patients fare worse against the general population in terms of 1-year and 5-year CVD risk projections when matched for the same conventional CVRFs (such as age, gender, total cholesterol, systolic BP etc), as shown in the US National Health and Nutrition Examination^9^.
5. Mineralocorticoid excess, oxidative stress and cellular inflammation are linked to the pathogenesis of plaque formation and rupture in CKD patients^10^.
6. An abnormal bone and mineral metabolic milieu accelerates CAD burden in CKD patients. It was shown that a lower serum 25-hydroxyvitamin D level is associated with a higher rate of myocardial infarction over a 10-year follow up^11^. Along the same line, increased fibroblast growth factor 23, a hormone that increases the rate of urinary phosphate excretion and inhibits renal 1,25(OH) vitamin D production, was independently associated with increased mortality in patients commencing dialysis^12^.

At the pathophysiologic level, the relationship between CKD and CAD, and how each entity promotes the acceleration of the other, is a complex interplay of factors involving hemodynamically mediated mechanisms, humoral and hormonal elements, and immune / inflammatory responses. In the presence of CKD, there is also emerging evidence that the pathology and manifestation of CVD in these patients are more varied and encompass the spectrum of heart failure, cardiac ischemia, atrial fibrillation, stroke, peripheral arterial disease, and sudden cardiac death. However, significant knowledge gaps still exist and efforts are ongoing in multiple trials and research undertaken by both the cardiology and renal communities to address these concerns^13^.

In 2013, the American Heart Association (AHA) issued a Scientific Statement on “Kidney Disease as a Risk Factor for Development of Cardiovascular Disease”^14^, maintaining a strong advocacy towards classifying CKD as an independent high-risk group for ASCVD development, and promoting the justification of stricter risk factor control than the general population in the CKD group.

## Revascularization Experiences in Chronic Kidney Disease

The linear relationship between worsening renal function and poorer survival and CVD events is clearly evident. Whether coronary revascularization affords protection in lowering such adverse outcomes remains unanswered. A number of studies have alluded that revascularization procedures, compared to medical therapy alone, appear to provide benefit. However, this is offset by graft failure, instent re-stenosis, repeat revascularization procedures and death in the CKD group when matched against individuals with normal or near normal renal function.

No robust evidence is as yet available to ascertain whether CKD patients who undergo revascularization have a definite survival advantage compared to CKD patients on medical therapy alone. Revascularization procedures are confounded by a different set of issues that confound survival curves, such as: (1) heightened bleeding risks resulting from the requirements for dual antiplatelet agents, as well as intra- and peri-procedural anticoagulation; (2) increased short term morbidity and mortality related to the interventions, especially in the selected group of CKD patients with acute coronary syndromes that necessitate urgent revascularization. Moreover, the necessary use of contrast agents during coronary angiography infrequently led to contrast induced nephropathy. This is paralleled by post-operative acute renal failure from renal ischaemia in CABG patients, with the subsequent need for dialysis, resulting in a higher risk for aggravating baseline renal status and escalating this subset of CKD patients into an even more tenuous CVD profile.

## Coronary Artery Bypass Surgery in CKD

**Figure 1.**
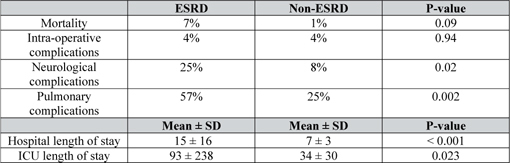
Univariate analysis of hospitalization outcome variables. J Card Surg 2004.

Contemporary studies specifically looking at CABG clinical outcomes in CKD patients are scant, and mainly consisted data pre-dating 2010. These studies showed that CKD patients that had clinically significant CAD, when revascularized via CABG, had better long-term outcomes compared to medical therapy alone^15^-^21^. However, end stage renal disease (ESRD) patients had worse peri-operative complication risks over non-ESRD counterparts (see Figure 1), even after adjusting for competing risk factors.

From the review^22^ analyzing the 2001 to 2006 data of 7 studies comparing off-pump CABG (OPCAB) viz-a-viz on-pump CABG (ONCAB), it was observed that there was a trend towards increased mortality in CKD patients undergoing ONCAB – 8% to 17% post-operative mortality in the ONCAB cohorts, versus 2% to 11% in the OPCAB cohort.

## Percutaneous Coronary Intervention for Acute Coronary Syndromes in CKD

Studies pertaining to PCI outcomes in CKD patients experiencing acute coronary syndromes are less common than those looking at revascularization of stable CAD. One of the recent studies was extracted from the ANIN registry^23^, which explored the prognostic value of renal impairment in STEMI patients that underwent acute PCI. The study concluded that when an estimated glomerular filtration rate (eGFR) of 60ml/min/1.73m^2^ was used as a cut-off, TIMI 3 flow was restored less frequently, in-hospital MACE was higher, 30-day mortality was increased, and major bleeding was also more common.

When stratified by creatinine clearance (as calculated by the Cockroft Gault formula), survival post primary PCI in CKD patients progressively got more dismal as CrCl worsened^24^. This was mirrored by higher mortality rates, at both 30-day (16% vs 1%, P < 0.0001) and 1-year (23% vs 3%, P < 0.0001), for the subsets that developed contrast-induced nephropathy.

Haemorrhagic complications and transfusion requirements were also more than doubled in CKD patients, as were severe restenoses (>70% diameter) and infarct-related artery reocclusions. Multivariate predictors of mortality also indicated a lower CrCl and left anterior descending artery infarct vessel as key contributors to worsened survival^24^.

## Percutaneous Coronary Intervention for Stable Coronary Artery Disease in CKD

There are more data and studies focused on the clinical outcomes of CKD patients with stable ischemic heart disease that underwent PCI.

As PCI became more prevalent and popular as a minimally invasive strategy in the 1990s, the clinical conundrum of subjecting CKD patients (commonly older with more comorbidities) to PCI as an alternative to the more widely accepted CABG (but higher surgical risk in the CKD setting), emerged as a hotly debatable issue. In a review of the limitations of PCI, Rubenstein et al studied the immediate and long-term clinical outcomes of CKD patients undergoing PCI through the mid 1990s^25^. The paper concluded that CKD patients had worse long term survival, lower procedural success rates, and greater in-hospital combined major CV events. Predictors of in-hospital MACE in the CKD group comprised shock, peripheral arterial disease (PAD), balloon angioplasty strategy, and unstable angina. Adverse outcomes were comparable between both non-dialysis and dialysis CKD cohorts. Notably, due to the nascency of coronary stents in that era, less than half of the PCI involved stenting.

The Mayo Clinic group, led by Best, stratified CKD patients according to varying degrees of renal dysfunction, and assessed the effect of the severity of renal insufficiency on PCI related MACE^26^. While the PCI of that era was balloon angioplasty-centric, the survival curves of the CKD cohorts, differentiated by creatinine clearance, maintained very early separation within months of the PCI. At the end of the 3 year follow-up period, it was observed that the lower the creatinine clearance, the higher the MACE rates and the poorer the survival. After adjusting for other co-existing cardiovascular risk factors, renal insufficiency remained a strong predictor of death and subsequent cardiac events in a dose-dependent fashion during and after PCI^26^.

As coronary stenting gained momentum and became the preferred PCI strategy of choice, a subsequent retrospective study in 2009 by Ki et al reinforced the observation that regardless of PCI strategies, a poorer baseline renal function was associated with a worse clinical outcome^27^.

However, patients in whom drug-eluting stents (DES) were implanted fared better than those with bare metal stents (BMS) in terms of all-cause mortality (13% vs 5%, P 0.025) and myocardial infarction post-PCI (10% vs 1.7%, P 0.003). Significantly, this study reviewed data of a predominantly Asian populace with CKD, and the results remained consistent with other earlier trials from America and Europe.

As drug-eluting stents evolved, more data emerged on their efficacy in the CKD subgroup. Drug eluting stents appeared to confer longer treatment longevity over bare metal stents by delaying instent restenosis, although target lesion revascularization rates were similar at the 6-month mark in a small observational study^28^. The EVENT registry^29^ analyzed data from almost 5000 CKD patients that underwent PCI with newer generation DES between 2004 to 2005, and noted that during the index hospitalization for PCI, there was a gradated increase in bleeding complications with decreasing CrCl, as well as increased death and MI. Notably, the group with the worst CrCl < 30ml/min also exhibited the highest rates of left main coronary disease, bifurcation lesions, as well as vein graft occlusions. However, despite these important observations, renal dysfunction was not associated with an increased risk of stent specific events, including stent thrombosis or clinical restenosis (i.e. target lesion revascularization).

Barthelemy et al compared the efficacies of DES to BMS head-to-head during PCI for CKD patients^30^. Cardiovascular death and MACE occurred less frequently in the DES group (both renal impaired and normal renal function), with no significant difference in target lesion revascularization (TLR) rates.

## PCI versus CABG in CKD

**Figure 2.**
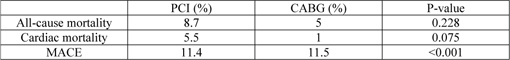
Univariate analysis of selected clinical outcomes in patients with eGFR<60ml/min/1.73m^2^ by management strategy used. J Korea Med Sci 2009.

Data derived from head-to-head comparisons between PCI and CABG in CKD populations are limited. A single centre experience comparing PCI and CABG for CKD patients demonstrated no mortality differences between the 2 groups^27^ (Figure 2). However, this study was retrospective in nature, and limited to a single centre with certain treatment bias.

A post-hoc subgroup analysis of the Arterial Revascularization Therapies Study (ARTS) compared a small group of renal insufficient patients (N = 142) that underwent either PCI or CABG, and tracked them over 5 years^31^. At the end of the 5-year period, there was no significant difference between the 2 groups in terms of mortality (14.5% in PCI vs 12.3% in CABG, P = 0.81), or combined end point of death, stroke or myocardial infarction. However, the incidence of MACE in the PCI group was higher than the CABG group, driven primarily by higher rates of repeat revascularization procedures in the stent group.

The above contrasted starkly against earlier observations made in the USRDS data review by Herzog et al^32^. For end stage CKD patients on dialysis, CABG was associated with a higher 2-year survival compared to PCI. In addition, stent outcomes were relatively worse if the CKD patients also had concomitant diabetes. However, this data was accumulated from the late 1990s when stenting were performed with predominantly bare metal stents.

**Figure 3.**
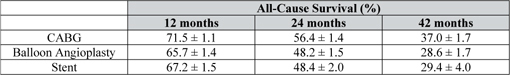
All-cause survival mean +/- SEM. Circ 2002.

In addition, two studies^33^, ^34^ showed that while CABG is superior to PCI in angina reduction and recurring revascularization, survival rates did not differ.

In the APPROACH study^35^, Hemmelgarn and colleagues reviewed retrospective data across the severity spectrum of CKD patients – namely, dialysis dependent CKD, nondialysis dependent CKD, and a control group (creatinine < 2.3mg/dL) – with respect to survival outcomes across 3 broad CAD management strategies (PCI, CABG, or medical therapy). The conclusion that CABG was associated with better prognosis in all categories of kidney function was consistent with previous observations. The study observed that PCI was associated with a lower mortality compared to medical therapy alone, though this finding was discordant in CKD patients who were non-dialysis dependent. The authors postulated that this anomaly might be attributable to acute-onchronic renal failure in the non-dialysis subgroup following PCI, which generally portends a worse CV outcome, as well as incomplete revascularization with PCI in the non-dialysis patient, owing to a more conservative use of angiographic contrast. However, the investigators also conceded that the limitations of this observational study included selection bias of patients who underwent revascularization procedures (generally the healthier patients), as well as a lack of details on medications, which prevented the characterization of the medical treatment arm. Nevertheless, it was a commendable effort that encompassed the analysis of data of a large population of patients (more than 40,000), and over a long horizon (8 year survival).

More recently, in the 2010 review paper^22^ by the Texas Heart Institute surveying the various revascularization options in patients with CKD, Ashrith et al established that advanced CKD patients, compared to those with milder renal disease (as defined by varying creatinine clearance values), are inherently at a higher risk for developing postoperative complications from CABG, such as operative death, stroke, prolonged mechanical ventilation, deep sternal wound infection, and new dialysis requirement. However, CABG remained the superior revascularization option in CKD patients with the best survival benefit, especially when the internal mammary artery is utilized as a bypass conduit.

Supporting CABG as the treatment option of choice in CKD patients with CAD, Herzog et al, extracted longitudinal data from the Medicare supported CKD patients undergoing coronary revascularization, and found that long term risks of death or combined death and ESRD were significantly lower after CABG, compared to PCI^36^.

Another registry in Canada looked at PCI data of over 4000 patients with CKD and multivessel CAD that underwent revascularization from 2008 to 2011, and observed that there was improved survival for dialysis dependent patients after CABG compared to PCI (80% vs 71%, P < 0.001), over a 3-year follow up period^37^.

These contradicting conclusions need to be viewed with caution, as these PCI vs CABG studies were mainly retrospective reviews and not powered to determine the true superiority of one revascularization strategy over the other. Moreover, selection bias plays a very important role in determining which subset of patients get selected for CABG, viz-a-viz those that get selected for PCI. Those patients with advanced CVD or debilitated general conditions will likely not be deemed suitable for bypass surgery, and if clinically warranted, any form of coronary revascularization will likely be in the option of PCI, driven by both patient and physician preference.

## Conclusion

The presence of CKD portends a poor prognosis for patients that develop clinically significant CAD. While the data remains conflicting when comparing the clinical efficacies of PCI versus CABG, the emergence of new generation drug-eluting stents and the utility of novel intraand postPCI pharmacotherapeutics (GPIIb/IIIa products, ticagrelor etc) shows promise to match, if not improve outcomes for patients that are not otherwise suitable to undergo surgery. More studies to compare CABG to DES PCI will be required to draw stronger conclusions between the two entities.

Presently, it is still best to be guided by holistic clinical assessment of the patient by the multidisciplinary heart team (comprising cardiologists and surgeons), taking into account the patient’s physical condition, co-morbidities, and most importantly his personal preference for coronary intervention, before deciding on the best course of management.
